# Evaluation of Non-Invasive Methods for (R)-[^11^C]PK11195 PET Image Quantification in Multiple Sclerosis

**DOI:** 10.3390/jimaging10020039

**Published:** 2024-01-31

**Authors:** Dimitri B. A. Mantovani, Milena S. Pitombeira, Phelipi N. Schuck, Adriel S. de Araújo, Carlos Alberto Buchpiguel, Daniele de Paula Faria, Ana Maria M. da Silva

**Affiliations:** 1 Faculdade de Medicina FMUSP, Universidade de Sao Paulo, Sao Paulo 05403-911, Brazil; dimitrimantovani@usp.br (D.B.A.M.); anammarques@usp.br (A.M.M.d.S.); 2 Laboratory of Nuclear Medicine (LIM 43), Department of Radiology and Oncology, Faculdade de Medicina FMUSP, Universidade de Sao Paulo, Sao Paulo 05403-911, Brazil; 3 Weill Cornell Medical College, New York, NY 10065, USA; 4 Graduate Program in Computer Science, Pontificia Universidade Catolica do Rio Grande do Sul PUCRS, Porto Alegre 90619-900, Brazil

**Keywords:** TSPO, neuroinflammation, kinetic modelling, quantification, image-derived input function

## Abstract

This study aims to evaluate non-invasive PET quantification methods for (R)-[^11^C]PK11195 uptake measurement in multiple sclerosis (MS) patients and healthy controls (HC) in comparison with arterial input function (AIF) using dynamic (R)-[^11^C]PK11195 PET and magnetic resonance images. The total volume of distribution (VT) and distribution volume ratio (DVR) were measured in the gray matter, white matter, caudate nucleus, putamen, pallidum, thalamus, cerebellum, and brainstem using AIF, the image-derived input function (IDIF) from the carotid arteries, and pseudo-reference regions from supervised clustering analysis (SVCA). Uptake differences between MS and HC groups were tested using statistical tests adjusted for age and sex, and correlations between the results from the different quantification methods were also analyzed. Significant DVR differences were observed in the gray matter, white matter, putamen, pallidum, thalamus, and brainstem of MS patients when compared to the HC group. Also, strong correlations were found in DVR values between non-invasive methods and AIF (0.928 for IDIF and 0.975 for SVCA, *p* < 0.0001). On the other hand, (R)-[^11^C]PK11195 uptake could not be differentiated between MS patients and HC using VT values, and a weak correlation (0.356, *p* < 0.0001) was found between VT^AIF^ and VT^IDIF^. Our study shows that the best alternative for AIF is using SVCA for reference region modeling, in addition to a cautious and appropriate methodology.

## 1. Introduction

Multiple sclerosis (MS) is a neurological disorder that causes severe damage to the brain and spinal cord through inflammatory and neurodegenerative processes [1,2]. MS features can be observed using imaging techniques such as magnetic resonance (MR) and positron emission tomography (PET), which can provide insightful information about the pathophysiology of MS patients [3].

Dynamic PET imaging using 18-kDa translocator protein (TSPO) tracers, such as (R)-[^11^C]PK11195, enables the evaluation of neuroinflammatory processes by the quantification of tracer distribution parameters, such as the total volume of distribution (VT) and the distribution volume ratio (DVR).

The most reliable method for fully quantitative PET analysis is compartmental modeling with a metabolite-corrected arterial plasma tracer concentration curve, also known as arterial input function (AIF) [4,5,6]. However, due to the requirement of arterial cannulation, sophisticated equipment, and experienced professionals, AIF extraction is considered an invasive and complex procedure. Non-invasive input functions, such as image-derived input functions (IDIF) and reference regions, approach [5,7].

Recent IDIF methods are commonly based on time-activity curves extracted from blood pool sites in the PET image, such as the aorta or the carotid arteries for images with limited field-of-view. Nevertheless, these methods are highly dependent on blood vessel morphology and very susceptible to motion and partial volume effects (PVE) [7,8,9,10,11,12,13]. Input functions from reference regions also provide an adequate approach to PET quantification based on time-activity curves from non-specific binding tissues [14,15,16,17]. Nevertheless, no reference region is available for TSPO tracer once innate immune cells are spread in the whole central nervous system (CNS), and virtually all CNS can be affected by MS pathology [3].

Alternatively, pseudo-reference regions can be identified by a supervised clustering algorithm (SVCA) that classifies voxels in PET images by their kinetic behavior [18,19,20]. SVCA assumes that the time-activity curve of any voxel in the brain can be described as a linear combination of four predefined kinetic classes extracted from this study population: gray matter, white matter, blood, and a high specific binding tissue. Using a multilinear regression approach, its output is a voxel cluster mask used as a pseudo-reference region for PET quantification [18].

IDIF from carotid arteries and SVCA methods have already been used in MS cohort studies [13,21,22,23,24,25]. However, there is a lack of MS studies that directly correlate these non-invasive methods with AIF. This study aims to evaluate non-invasive methods (IDIF and SVCA) for (R)-[^11^C]PK11195 uptake measurement in PET images of relapsing-remitting MS (RRMS) patients and healthy controls (HC) in a direct comparison to AIF.

## 2. Materials and Methods

### 2.1. Study Cohort

This study is a retrospective analysis of a cross-sectional study that was approved by our local institutional ethics committees (registration number: 2.451.027) in accordance with the 1964 Declaration of Helsinki [26]. All image data were already anonymized to preserve participant confidentiality. All images were acquired at the Nuclear Medicine Center of the Hospital das Clinicas at the University of São Paulo, USP [26,27]. Shortly, (R)-[^11^C]PK11195 PET/MR images from 24 RRMS patients and 16 HC were evaluated. Table 1 shows the demographic data. Further information about the participants’ inclusion and exclusion criteria can be found in de Souza et al., 2021 and Pitombeira et al., 2022 [26,27].

### 2.2. Image Acquisition

(R)-[^11^C]PK11195 60-min dynamic PET images were acquired in a hybrid PET/MR 3 T system (SIGNA, General Electric Healthcare, Milwaukee, WI, USA) with approximately 4.0 mm of spatial resolution [26], simultaneously with a 1 min intravenous injection containing 385.54 ± 0.47 MBq of tracer activity. Arterial blood samples were manually obtained throughout the 60 min dynamic acquisition at 1 s, 10 s, 30 s, 45 s, 60 s, 90 s, 2 min, 3 min, 5 min, 10 min, 20 min, 30 min, 45 min, and 60 min postinjection. In addition, blood samples collected at 20, 45, and 60 min postinjection were used for metabolite analysis. Further information about the blood analysis is described by de Souza et al., 2021 [27].

### 2.3. Image Reconstruction

The 60 min PET list mode data were reconstructed in 21 frames (6 × 10 s, 2 × 30 s, 3 × 60 s, 2 × 120 s, 2 × 180 s, 3 × 300 s, and 3 × 600 s) using a 3D OSEM algorithm (2 iterations and 28 subsets) with time-of-flight information, resolution modeling, and using the GE atlas-based method for attenuation correction. After reconstruction, each frame of the dynamic PET image was smoothed with a 3 mm Gaussian filter for noise reduction. Three-dimensional MR images were acquired with T1-weighted fast spoiled gradient recalled-echo sequences (TR = 7.6 ms, TE = 3.1 ms, TI = 600 ms, flip angle = 8° slice thickness = 1 mm) using a 24-channel Head Neck Unit coil [26].

### 2.4. Image Processing

PET/MR image analysis was performed in PMOD 4.0 (PMOD Technologies Ltd., Zurich, Switzerland). Dynamic PET images were interframe motion-corrected using a rigid registration approach with the average of 11 first frames as the reference frame and co-registered with the respective T1-weighted MR images in the PNEURO tool. Time-activity curves from the cortical gray matter (brain and cerebellum), white matter, caudate nucleus, putamen, pallidum, thalamus, whole cerebellum, and brainstem VOIs were extracted using N30R83 Hammers-Atlas [28,29].

### 2.5. Input Functions: Extraction

Circular regions of interest (ROI) of 4 mm in diameter were placed in 4 consecutive slices over the carotid arteries’ C4 portion on the averaged 7 first frames (90 s) from PET registered to MRI [12]. These ROIs were dilated in 2 voxels, generating an enlarged volume of interest (VOI) related to the radioactivity spillover to adjacent tissues. Then, the enlarged VOI was dilated by 4 voxels to obtain a background VOI. The VOIs were applied to the dynamic data to obtain the carotid, adjacent tissues, and background time-activity curves. PVC was performed on IDIF data using the external PVC tool with a 4 × 4 × 4 mm^3^ point spread function (PSF) in PMOD. Finally, a metabolite correction was performed using population-based data from a previous study [27], which investigated the (R)-[^11^C]PK11195 metabolization in MS patients and healthy subjects in a Brazilian cohort. 

Pseudo-reference regions were obtained using a four-tissue-based SVCA (SVCA4) [18,19,20]. The first step for the SVCA4 model construction was a whole-brain framewise normalization of the dynamic PET data of each subject, which was used to extract pre-defined kinetic classes. Each voxel value in the PET data were subtracted from the mean whole-brain frame’s value and divided by the whole-brain frame’s standard deviation. Gray matter, white matter, and blood pool classes were extracted from the HC images, while the high specific binding class was extracted from MS patients’ thalamus [18]. Carotid arteries from IDIF were used for the blood pool class [30]. Quality control steps were performed during extracting the pseudo-reference region, such as the leave-one-out procedure for SVCA4 construction and checking if the clusters’ time-activity curves had high activity peaks and fast washouts [30]. We also investigated the effect of excluding the first 20 min from the PET data on the SVCA4 results, aiming to reduce time-consuming acquisitions due to dynamic acquisitions.

### 2.6. PET Quantification

Logan graphical models were used to estimate the pharmacokinetic parameters related to the (R)-[^11^C]PK11195 PET distribution [31,32]. For AIF and IDIF methods, the Logan model based on a blood input function (Logan Plot) was used to estimate VT in target tissues in the last 40 min of the PET acquisition (t* = 20 min). Clusters from SVCA4 were used as input for the Logan Reference analysis for DVR estimation in target tissues (t* = 20 min).

The fitting performance of the Logan graphical analysis for each input function was assessed through the determination coefficient (R^2^), since Akaike’s information criterion cannot be applied due to the presence of the noisy term on both sides of the model equation. To compare all input function methods, DVR values were calculated for AIF and IDIF as the ratio between the target tissue VT and the pseudo reference region VT. Bland–Altman analysis and linear regressions were applied to compare the parameters from the non-invasive methods with the AIF. Correlations were assessed by the Pearson coefficients. The agreement between the VT and DVR parameters was also evaluated in the correlation analysis.

### 2.7. Statistical Analysis

Student’s *t*-tests with 95% confidence and unassumed equal variances were applied to identify whether there were age differences between groups, and chi-square tests were also used to assess differences concerning sex. Generalized linear models (GLM) adjusted for age and sex were used on VT and DVR values to evaluate whether the quantification methods could identify (R)-[^11^C]PK11195 binding differences in selected brain regions between the MS and HC groups. All statistics were performed using SPSS (IBM SPSS Statistics for Windows, version 21.0; Armonk, NY, USA: IBM Corp.). All figures were created using GraphPad Prism (GraphPad Prism version 10.0.0 for Windows, GraphPad Software, Boston, MA, USA).

## 3. Results

### 3.1. Input Functions: Extraction and Corrections

The PVC method enabled the partial recovery of the carotid spill-over, resulting in an overall increase of approximately 109% in the IDIF area under the curve (AUC). It is important to emphasize that these results were obtained using the geometric transfer matrix method in PMOD, which is non-dependent on blood data, thus making the process completely non-invasive.

The pseudo-reference regions were extracted using a leave-one-out procedure, avoiding the bias of testing the model on the data used to build it. Thus, a unique SVCA4 model was constructed and applied for each subject without seeing its data. The results of the pseudo-reference region kinetic analysis can be found in the Supplementary Material (Figures S1 and S2).

### 3.2. Image Quantification

All quantification models showed R^2^ values equal to or higher than 0.99 for AIF, IDIF, and SVCA4.

Table 2 shows the VT mean, standard deviation, and *p*-values using the AIF (VT^AIF^) and IDIF (VT^IDIF^) for the HC and MS groups. For almost all brain regions, slightly higher VT^AIF^ and VT^IDIF^ values were observed in the MS cohort when compared to the HC group; however, the statistical analysis (GLM) showed that there are no significant differences in (R)-[^11^C]PK11195-VT values between the groups. Additional Student’s *t*-tests were performed without age and sex corrections for VT^AIF^ and VT^IDIF^, as no statistical difference was found.

**Figure 1:**
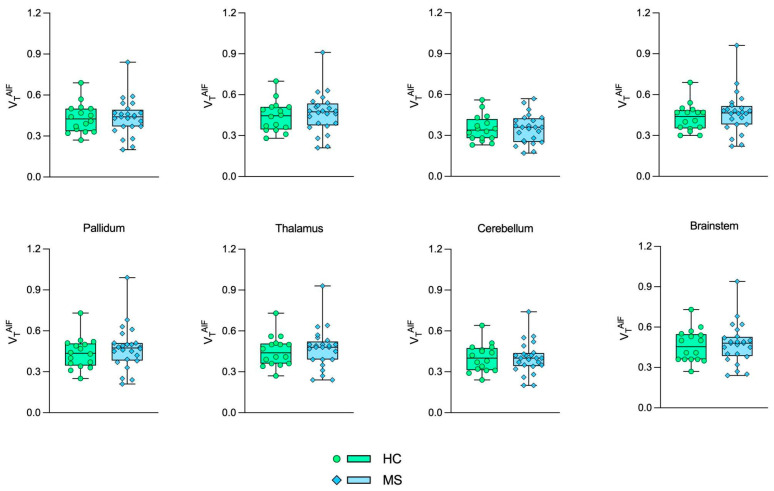
Boxplot representation of VT^AIF^. Green boxes with circles represent the VT^AIF^ values in the HC group, and blue boxes with diamonds represent the VT^AIF^ values in the MS group. HC: Healthy control. MS: Multiple sclerosis.

**Figure 2:**
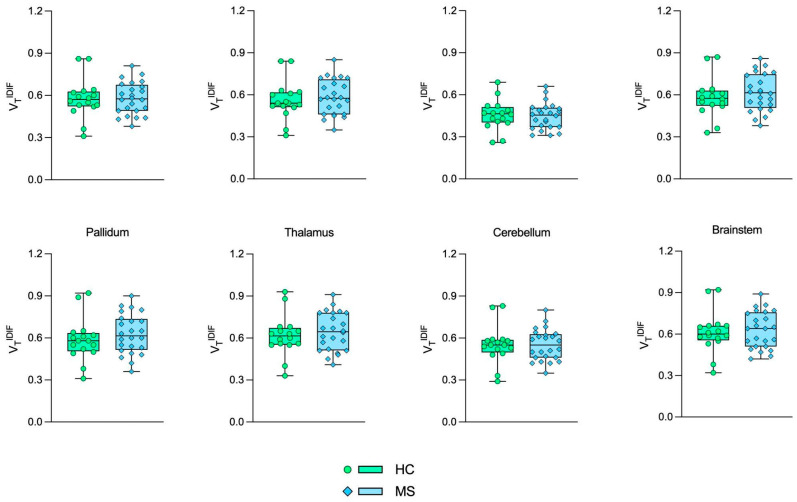
Boxplot representation of VT^IDIF^. Green boxes with circles represent the VT^IDIF^ values in the HC group, and blue boxes with diamonds represent the VT^IDIF^ values in the MS group. HC: Healthy control. MS: Multiple sclerosis.

Figure 1 and Figure 2 show the boxplot representation of VT^AIF^ and VT^IDIF^ parameters in MS and HC groups, respectively. Despite the higher deviation from the mean for VT^IDIF^ in the HC group and the overall VT overestimation by IDIF in comparison to AIF, the VT distribution was very similar among the groups.

**Figure 3:**
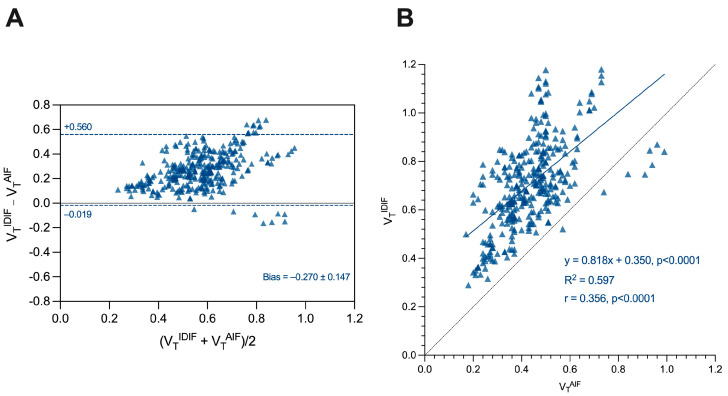
Bland–Altman (**A**) and linear regression analysis (**B**) of VT^IDIF^ and VT^AIF^.

Figure 3 shows the Bland-Altman (3A) and linear regression plots (3B) from the VT comparison between AIF and IDIF. The Bland–Altman analysis showed a bias equal to 0.276 ± 0.147 in VT values, highlighting the VT^IDIF^ overestimation when compared to VT^AIF^. The linear regression resulted in R^2^, slope, and intercept values equal to 0.596, 0.818 (*p* < 0.0001), and 0.350, respectively. A weak correlation was found between the VT measurements (0.356, *p* < 0.0001).

Table 3 shows the DVR mean, standard deviation, and *p*-values using the AIF (DVR^AIF^), IDIF (DVR^IDIF^), and SVCA4 (DVR^SVCA4^) for the HC and MS groups (Tables S1 and S2 in the Supplementary Material show no sex differences in the analysis). Higher DVR values were found in the MS group in comparison to the control group, and GLM tests identified significant differences in the same brain regions for all quantification methods. The DVR differences were observed in white matter, putamen, pallidum, and thalamus. The respective *p*-values are shown in Table 3.

**Figure 4:**
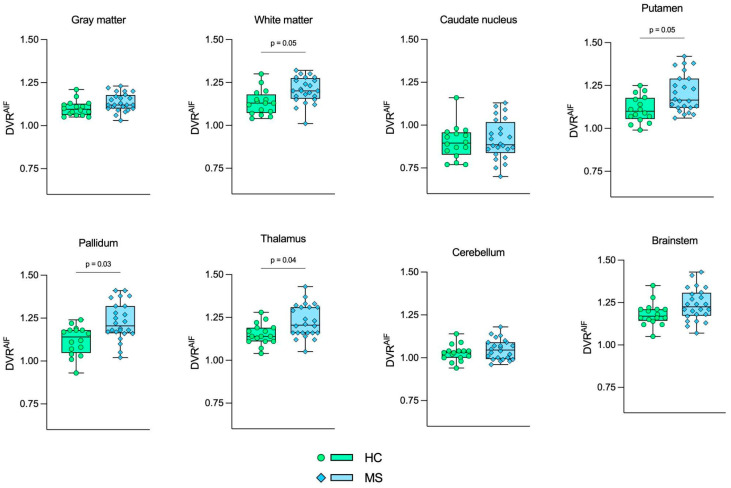
Boxplot representation of DVR^AIF^. Green boxes with circles represent the DVR^AIF^ values in the HC group, and blue boxes with diamonds represent the DVR^AIF^ values in the MS group. The *p*-values from GLM tests are shown, respectively. HC: Healthy control. MS: Multiple sclerosis.

**Figure 5:**
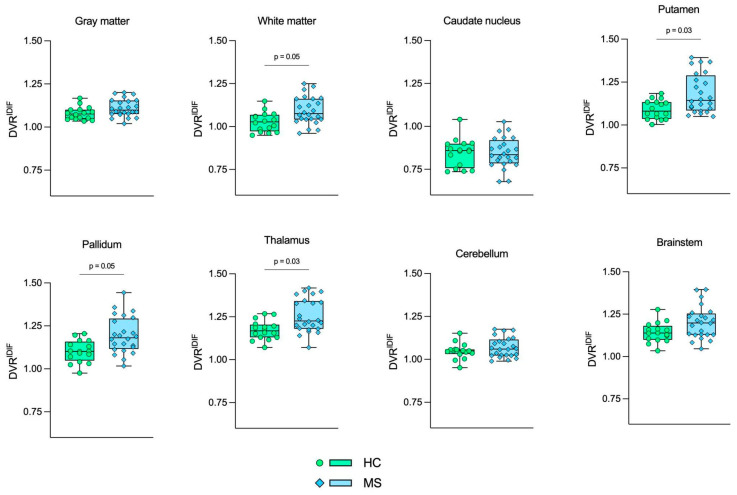
Boxplot representation of DVR^IDIF^. Green boxes with circles represent the DVR^IDIF^ values in the HC group, and blue boxes with diamonds represent the DVR^IDIF^ values in the MS group. The *p*-values from GLM tests are shown, respectively. HC: Healthy control. MS: Multiple sclerosis.

**Figure 6:**
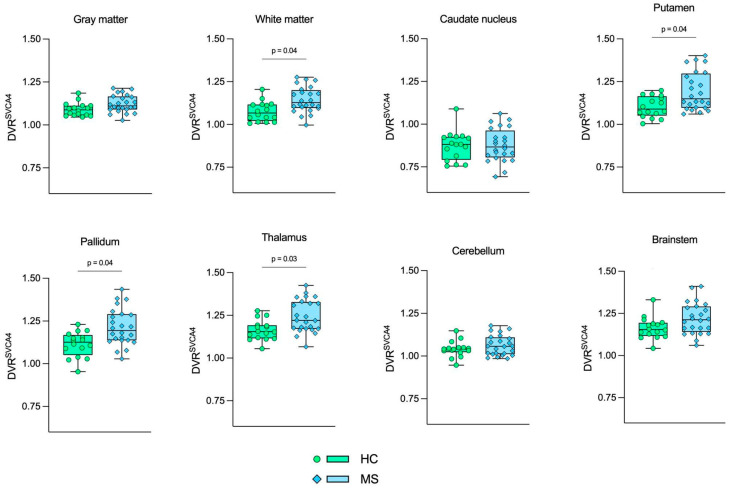
Boxplot representation of DVR^SVCA4^. Green boxes with circles represent the DVR^SVCA4^ values in the HC group, and blue boxes with diamonds represent the DVR^SVCA4^ values in the MS group. The *p*-values from GLM tests are shown, respectively. HC: Healthy control. MS: Multiple sclerosis.

**Figure 7:**
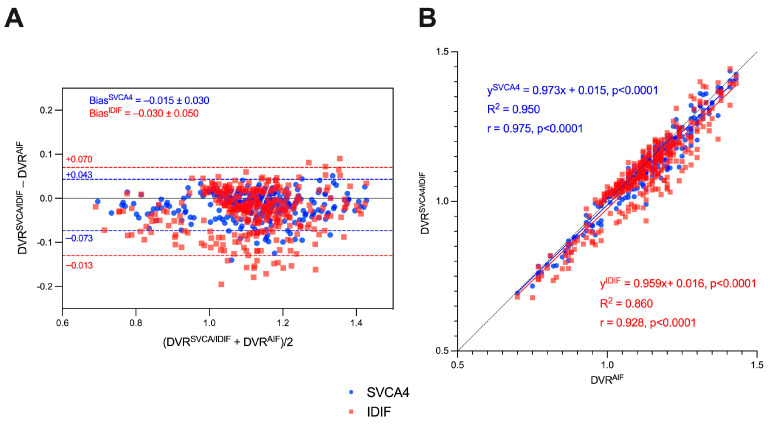
Bland–Altman (**A**) and linear regression analysis (**B**) of DVR^AIF^, DVR^IDIF^, and DVR^SVCA4^ (IDIF in red and SVCA4 in blue).

Figure 4, Figure 5 and Figure 6 show the boxplot representation of DVR^AIF^, DVR^IDIF^, and DVR^SVCA4^ parameters in both HC and MS groups, respectively. These figures show a strong similarity between the DVR distribution among all quantification methods, despite the fact that IDIF and SVCA4 underestimated the (R)-[^11^C]PK11195-DVR values in both groups in comparison to AIF. Such similarities and underestimations in DVR values are highlighted by Figure 7. The Bland–Altman (7A) analysis showed a bias equal to −0.030 ± 0.050 for IDIF and −0.015 ± 0.030 for SVCA4. The linear regression resulted in R^2^, slope, and intercept values of 0.860, 0.959 (*p* < 0.0001), and 0.016 for IDIF, and 0.950, 0.973 (*p* < 0.0001), and 0.015, respectively, for SVCA4.

**Figure 8:**
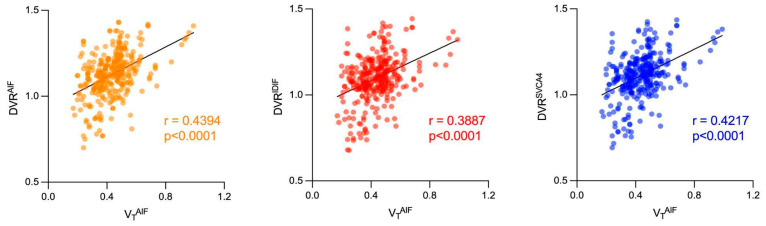
Correlation analysis between VT^AIF^ and DVR^AIF^ (orange), DVR^IDIF^ (red), and DVR^SVCA4^ (blue).

The results of the correlation analysis of VT and DVR parameters are shown in Figure 8. A moderate correlation between DVR and VT^AIF^ was found for DVR^AIF^ (0.4394, *p* < 0.0001) and DVR^SVCA4^ (0.4217, *p* < 0.0001), while a weak correlation was found for DVR^IDIF^ (0.3887, *p* < 0.0001).

**Figure 9:**
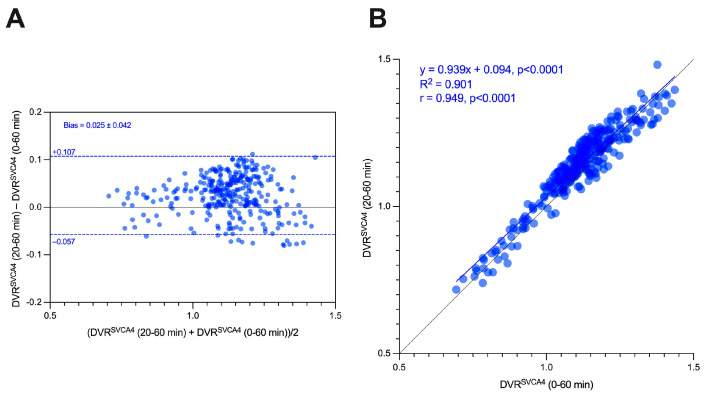
Bland–Altman (**A**) and linear regression analysis (**B**) of DVR^SVCA4^ (0–60 min) and DVR^SVCA4^ (20–60 min).

Figure 9 shows the Bland–Altman (9A) and linear regression (9B) analysis between the DVR^SVCA4^ values quantified using the full image (0–60 min) and the reduced image (20–60 min). It is noted that both methods provided very similar results, with a bias equal to 0.025 ± 0.042 and the parameters from the linear regression, R2, slope, and intercept equal to 0.901, 0.939 (*p* < 0.0001), and 0.015. Finally, a strong correlation was found between the two variables (0.975, *p* < 0.0001).

## 4. Discussion

This study evaluated two fully non-invasive approaches (IDIF and SVCA4) to provide reliable kinetic analysis in a cohort of patients with MS in comparison with arterial blood sampling. Our results show that SVCA4, together with the Logan Reference analysis (t* = 20 min), is the most reliable alternative for (R)-[^11^C]PK11195 kinetic modeling in both healthy subjects and MS patients, providing DVR results that could identify statistically significant (R)-[^11^C]PK11195 uptake differences in multiple subcortical regions and in the white matter, even correcting for age and sex (Table 3). Also, the pseudo-reference region extraction have not been affected significantly by excluding the initial 20 min data from the predefined kinetic classes, showing that the SVCA4 method is applicable for shorter (R)-[^11^C]PK11195-PET acquisitions.

Our results are consistent with other studies in the literature that assessed the (R)-[^11^C]PK11195 pharmacokinetics with DVR, including patients with different MS phenotypes [21,22,23,24]. In corroboration, the DVR and the non-dispensable binding potential (DVR-1), quantified through linearization analysis, are the most used parameters in MS studies, followed by the VT quantification [12,13,25]. Undoubtedly, blood sampling is the most reliable method for tracer concentration measurement in the patient’s bloodstream; however, the high variability of VT values in both controls and MS patients and the poor correlation between VT^AIF^ and VT^IDIF^ found in our results suggest that VT quantification by linearization methods using blood-derived input functions, even with arterial blood data, may not be the best alternative for neuroinflammation assessment in MS patients. These results are supported by the Pitombeira et al. [26] study, where no significant (R)-[^11^C]PK11195-VT differences were found in the same relapsing-remitting MS patients, but they also included primary and secondary progressive MS phenotypes in the analysis. Several factors may be related to these findings, such as the (R)-[^11^C]PK11195-specific binding to plasma proteins and endothelial cells and also errors associated with IDIF obtention. But because a very similar DVR distribution was found for all input function methods (AIF, IDIF, and SVCA4), we suppose that the differences in the results are much more linked to the analyzed parameter than the input function method used.

Our results show that when normalized for SVCA4-derived pseudo-reference regions, the (R)-[^11^C]PK11195 uptake analysis becomes more reliable, as suggested by a recent review study about TSPO quantification [33]. In the study of Kang et al. [12], the authors investigated the behavior of VT and DVR parameters in a test-retest study to identify the most robust method for TSPO-PET analysis in MS chronic lesions. They concluded that the VT quantified through the Logan graphical analysis using a PVE-uncorrected IDIF should be the best non-invasive alternative, a deviation from the present study. Some critical points may be responsible for the differences in the results from the present study in comparison with Kang et al. [12], such as the carotid delineation and the pseudo-reference region extraction, which both are essential steps to quantify the tracer distribution and are present in both studies.

Our carotid segmentation is based on Kang and collaborators’ methodology, with a circular ROI in the C4 portion of both left and right carotids. However, we aimed to include volumetric radioactivity measurements by placing consecutive circular ROIs, mainly to account for the radioactivity spillover in adjacent tissues and background [12]. Furthermore, these modifications provided an average 109% increase in input function AUC through the geometric transfer matrix method, which should enhance the robustness of the IDIF analysis. In addition, all PVC analyses can be considered non-invasive since there is no requirement for blood data to rescale the input curve.

A processing step directly related to the DVR and BP_ND_ values is pseudo reference region extraction. The supervised clustering methodology is used to classify low-binding gray matter voxels based on their kinetic behavior through a multilinear regression analysis, and its result is highly dependent upon the predefined kinetic classes used to fit the constant weights. Therefore, possibly the leading cause of the differences between the studies is in obtaining these predefined curves. In the present study, the kinetic classes were extracted from this study population using several quality control processes, such as the leave-one-out method and evaluations of previously mentioned kinetic characteristics. The leave-one-out methodology prevents the multilinear regression from being contaminated with the data being processed, thus testing the generalizability of the SVCA4 model [30]. Also, kinetic aspects such as high peak activity concentrations and fast washouts are desirable when using reference-tissue modeling approaches, and therefore such characteristics should be checked through kinetic rate constants (K1, k2, K1/k2) from full compartmental modeling analysis [30,33]. Such information was assessed in the SVCA4-derived pseudo-reference regions in our study using the reversible two-tissue compartment model (2T4k) with the AIF. The results can be found in the supplementary material (Figure S3).

A limiting factor in our data analysis, in comparison with Kang’s investigation [12], is that the variability between (R)-[^11^C]PK11195 scan sessions (test-retest) could not be assessed. As such, information about the stability of the VT and DVR parameters between scan sessions could not be compared in our study cohort. Further details on the (R)-[^11^C]PK11195 kinetic analysis stability in MS studies can be found in the study of Kang et al. [12].

Another very important factor for the reliability and accuracy of tracer quantification is the image reconstruction step, mainly for dynamic series, which are remarkable for noisy frames and possibly affected by patient motion. The standard 3D OSEM algorithm, which was applied in this study, does not account for motion effects during the projection data correction steps, such as attenuation correction, thus affecting the image resolution and the reliability of the quantitative data extracted from the reconstructed images [34]. In addition, PVC techniques for the whole brain were not applied to our (R)-[^11^C]PK11195-PET images, which may be a future improvement in the reconstruction of our acquisition data through iterative methods with a priori information using MR images, together with motion compensation, which can greatly improve the noise reduction and quantification accuracy [35,36].

Finally, reducing the dynamic scan time for (R)-[^11^C]PK11195-PET studies can offer several benefits, including increased patient comfort, fewer movement artifacts, time savings, and still being able to perform reliable tracer kinetic modeling using the Logan Reference analysis. This could be a promising approach for future studies investigating innate immune cell activation in neurological disorders, mainly in MS.

## 5. Conclusions

In conclusion, our study shows that the best alternative for invasive (R)-[^11^C]PK11195 quantification methods in MS patients is the SVCA4, in addition to a cautious and appropriate methodology. However, new IDIF methods should be applied to (R)-[^11^C]PK11195-PET images in order to find a better agreement between the quantitative parameters. Finally, as the aim of this study was only to compare the quantification output from non-invasive input functions and AIF, further studies may also aim to evaluate the feasibility of generating (R)-[^11^C]PK11195-DVR parametric maps and their respective readouts by nuclear physicians to assess the potential clinical applications of TSPO-PET.

## Figures and Tables

**Table 1:** Demographic information from study cohort.

	HC	RRMS	*p*-Value
Age (years)	41.8 ± 12.7	35.0 ± 7.6	0.06 ^&^
Sex (F/M)	11/5	16/8	0.73 ^#^
Education (years)	14.2 ± 3.9	13.7 ± 3.6	0.70 ^&^
EDSS (range)	-	1.0–6.0	-
Disease duration (years)	-	9.1 ± 6.4	-
Number of relapses	-	7.0 ± 8.0	-
Use of DMT (Y/N)	-	22/2	-

^&^: Student’s *t*-test. ^#^: Fischer’s exact test. HC: Healthy control. RRMS: Relapsing-Remitting Multiple Sclerosis. F: Female. M: Male. Y: Yes. N: No. EDSS: Expanded Disability Status Scale. DMT: Disease-Modifying Therapy.

**Table 2:** VT values for the blood-based input functions.

	AIF ^a^			IDIF ^a^		
	HC ^b^ (*n* = 16)	MS ^b^ (*n* = 24)	*p*-Value ^c^	HC ^b^ (*n* = 16)	MS ^b^ (*n* = 24)	*p*-Value ^c^
Gray matter	0.43 ± 0.11	0.44 ± 0.13	0.86	0.58 ± 0.14	0.58 ± 0.12	0.24
White matter	0.44 ± 0.11	0.46 ± 0.15	0.80	0.56 ± 0.14	0.58 ± 0.13	0.23
Caudate nucleus	0.35 ± 0.09	0.35 ± 0.11	0.61	0.46 ± 0.11	0.45 ± 0.09	0.41
Putamen	0.43 ± 0.10	0.46 ± 0.15	0.77	0.58 ± 0.14	0.62 ± 0.13	0.17
Pallidum	0.44 ± 0.12	0.47 ± 0.16	0.77	0.59 ± 0.15	0.63 ± 0.14	0.18
Thalamus	0.45 ± 0.11	0.47 ± 0.15	0.84	0.62 ± 0.15	0.65 ± 0.14	0.19
Cerebellum	0.40 ± 0.10	0.40 ± 0.12	0.88	0.55 ± 0.14	0.55 ± 0.11	0.23
Brainstem	0.46 ± 0.12	0.47 ± 0.15	0.79	0.61 ± 0.15	0.63 ± 0.13	0.34

^a^: Logan Plot. ^b^: mean ± standard deviation. ^c^: Generalized linear model adjusted for age and sex (95%). AIF: Arterial input function. IDIF: Image-derived input function. HC: Healthy control. MS: Multiple sclerosis patients.

**Table 3:** DVR values for each quantification method.

	AIF			IDIF			SVCA4		
	HC ^a^ (n = 16)	MS ^a^ (n = 24)	*p*-Value ^b^	HC ^a^ (n = 16)	MS ^a^ (n = 24)	*p*-Value ^b^	HC ^a^ (n = 16)	MS ^a^ (n = 24)	*p*-Value ^b^
Gray matter	1.10 ± 0.04	1.13 ± 0.05	0.12	1.07 ± 0.03	1.11 ± 0.05	0.08	1.09 ± 0.03	1.12 ± 0.05	0.09
White matter	1.13 ± 0.07	1.20 ± 0.07	0.05 *	1.02 ± 0.05	1.09 ± 0.08	0.05 *	1.07 ± 0.05	1.14 ± 0.07	0.04 *
Caudate nucleus	0.90 ± 0.09	0.91 ± 0.11	0.77	0.84 ± 0.08	0.84 ± 0.08	0.95	0.87 ± 0.08	0.87 ± 0.09	0.90
Putamen	1.11 ± 0.07	1.20 ± 0.11	0.05 *	1.09 ± 0.05	1.18 ± 0.11	0.03 *	1.10 ± 0.06	1.19 ± 0.11	0.04 *
Pallidum	1.11 ± 0.08	1.23 ± 0.11	0.03 *	1.10 ± 0.06	1.19 ± 0.10	0.05 *	1.11 ± 0.07	1.21 ± 0.10	0.04 *
Thalamus	1.15 ± 0.06	1.22 ± 0.09	0.04 *	1.17 ± 0.05	1.25 ± 0.09	0.03 *	1.16 ± 0.05	1.24 ± 0.09	0.03 *
Cerebellum	1.02 ± 0.04	1.04 ± 0.05	0.46	1.04 ± 0.04	1.06 ± 0.05	0.51	1.03 ± 0.04	1.06 ± 0.05	0.44
Brainstem	1.18 ± 0.06	1.23 ± 0.09	0.09	1.14 ± 0.05	1.20 ± 0.09	0.13	1.16 ± 0.06	1.21 ± 0.09	0.12

^a^: mean ± standard deviation. ^b^: Generalized linear model adjusted for age and sex (95%). AIF: Arterial input function. IDIF: Image-derived input function. HC: Healthy control. MS: Multiple sclerosis. *: *p*-Value<0.05.

## Data Availability

The data supporting the findings of these studies are available from the corresponding author upon reasonable request.

## Supplementary Materials

The following supporting information can be downloaded at: https://www.mdpi.com/article/10.3390/jimaging10020039/s1, Figure S1: (R)-[11C]PK11195 kinetics in SVCA4-derived pseudo reference region assessed by the 2T4kvB model using AIF. Light gray boxes represent the HC group while dark gray boxes represent the MS group, Figure S2: (R)-[11C]PK11195-VT in SVCA4-derived pseudo reference regions measured with the Logan Plot using AIF and IDIF. Light gray boxes represent the HC group while dark gray boxes represent the MS group, Figure S3: (R)-[11C]PK11195 time-activity curves in SVCA4-derived pseudo reference regions in both HC and MS groups; Table S1: Descriptive statistics of DVR^AIF^ values separated by sex in healthy controls, Table S2: Descriptive statistics of DVRAIF values separated by sex in MS patients.
