# Supplementary material for: Evaluation of Non-Invasive Methods for (R)-[11C]PK11195 PET Image Quantification in Multiple Sclerosis
Source: J Imaging. 2024 Jan 31;10(2):39. doi: 10.3390/jimaging10020039 (PMC10889702; doi:10.3390/jimaging10020039)
Supplement: Supplementary file 1 [file jimaging-10-00039-s001.zip › jimaging-2765021-supplementary.pdf]

Supplementary material of “Evaluation of non-invasive methods for (R)-[^{11}C]PK11195 PET images quantification in multiple sclerosis”

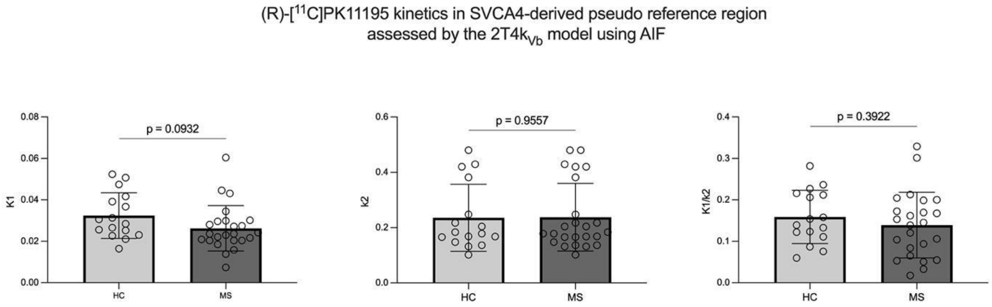

Figure S1. (R)-[11C]PK11195 kinetics in SVCA4-derived pseudo reference region assessed by the 2T4kvB model using AIF. Light gray boxes represent the HC group while dark gray boxes represent the MS group.

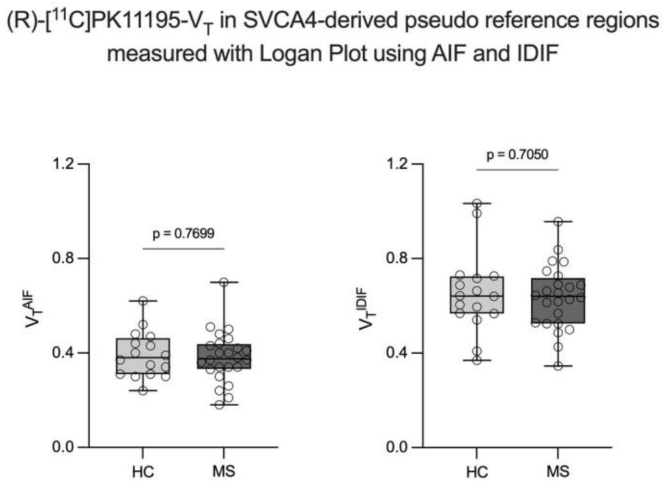

Figure S2. (R)-[11C]PK11195-V_T in SVCA4-derived pseudo reference regions measured with the Logan Plot using AIF and IDIF. Light gray boxes represent the HC group while dark gray boxes represent the MS group.

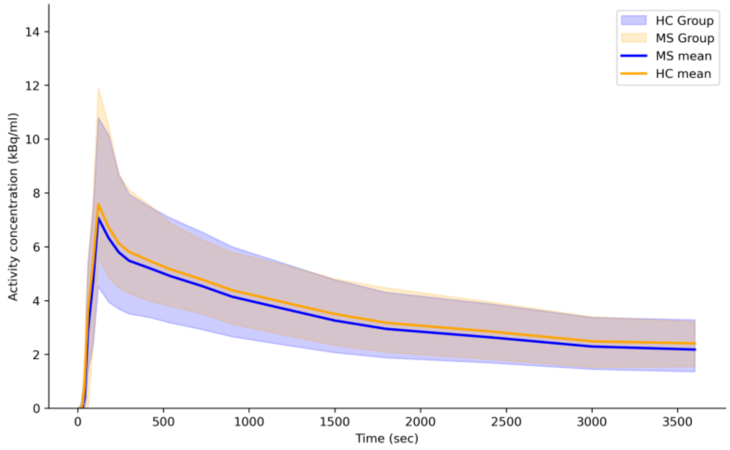

Figure S3. (R)-[11C]PK11195 time-activity curves in SVCA4-derived pseudo reference regions in both HC and MS groups.

Table S1. Descriptive statistics of DVR^{AIF} values separated by sex in healthy controls.

	Healthy controls															
	Gray matter		White matter		Caudate nucleus		Putamen		Pallidum		Thalamus		Cerebellum		Brainstem	
	M	F	M	F	M	F	M	F	M	F	M	F	M	F	M	F
Number	5	11	5	11	5	11	5	11	5	11	5	11	5	11	5	11
Minimum	1.05	1.05	1.04	1.07	0.82	0.77	1.02	0.99	1.03	0.93	1.11	1.04	0.97	0.94	1.05	1.12
Maximum	1.17	1.21	1.25	1.3	0.94	1.16	1.21	1.25	1.22	1.24	1.28	1.24	1.08	1.14	1.28	1.35
Range	0.12	0.16	0.21	0.23	0.12	0.39	0.19	0.26	0.19	0.31	0.17	0.2	0.11	0.2	0.23	0.23
Mean	1.092	1.105	1.106	1.147	0.884	0.9082	1.094	1.12	1.13	1.115	1.166	1.147	1.032	1.026	1.164	1.191
Std. Deviation	0.04764	0.04435	0.08792	0.06544	0.04393	0.1169	0.07301	0.08025	0.08832	0.08892	0.06731	0.06198	0.03962	0.05353	0.08735	0.06188
Std. Error of Mean	0.02131	0.01337	0.03932	0.01973	0.01965	0.03526	0.03265	0.0242	0.0395	0.02681	0.0301	0.01869	0.01772	0.01614	0.03906	0.01866
Coefficient of variation	4.363%	4.012%	7.949%	5.704%	4.970%	12.88%	6.673%	7.165%	7.816%	7.978%	5.772%	5.403%	3.839%	5.215%	7.504%	5.196%
p-value	0.609		0.384		0.559		0.539		0.755		0.613		0.819		0.622	

Table S2. Descriptive statistics of DVR^{AIF} values separated by sex in MS patients.

	MS patients															
	Gray matter		White matter		Caudate nucleus		Putamen		Pallidum		Thalamus		Cerebellum		Brainstem	
	M	F	M	F	M	F	M	F	M	F	M	F	M	F	M	F
Number	8	16	8	16	8	16	8	16	8	16	8	16	8	16	8	16
Minimum	1.03	1.06	1.1	1.01	0.7	0.81	1.08	1.06	1.05	1.02	1.05	1.12	0.98	0.96	1.07	1.11
Maximum	1.2	1.23	1.3	1.32	1.11	1.13	1.42	1.38	1.41	1.41	1.33	1.43	1.14	1.18	1.41	1.43
Range	0.17	0.17	0.2	0.31	0.41	0.32	0.34	0.32	0.36	0.39	0.28	0.31	0.16	0.22	0.34	0.32
Mean	1.124	1.141	1.199	1.209	0.8763	0.935	1.189	1.214	1.216	1.238	1.205	1.239	1.041	1.052	1.2	1.256
Std. Deviation	0.05528	0.05097	0.06978	0.07864	0.145	0.0994	0.121	0.1086	0.1188	0.1091	0.1052	0.09059	0.06198	0.05913	0.109	0.08253
Std. Error of Mean	0.01954	0.01274	0.02467	0.01966	0.05127	0.02485	0.04278	0.02716	0.042	0.02727	0.03718	0.02265	0.02191	0.01478	0.03854	0.02063
Coefficient of variation	4.919%	4.466%	5.821%	6.506%	16.55%	10.63%	10.18%	8.945%	9.767%	8.815%	8.726%	7.309%	5.952%	5.621%	9.085%	6.570%
p-value	0.466		0.755		0.326		0.622		0.678		0.445		0.694		0.224	
